# Healers that hurt: a scoping review of media reports of cases of rape in healthcare settings

**DOI:** 10.1186/s40359-024-01721-w

**Published:** 2024-04-16

**Authors:** Adaobi Margaret Okonji, Adeyinka G. Ishola, Love Bukola Ayamolowo, Omowumi M. Femi-Akinlosotu, Boladale Mapayi, Morenike Oluwatoyin Folayan

**Affiliations:** 1 Girls Club, Department of Mental Health, Obafemi Awolowo University, Ile-Ife, Nigeria; 2 Department of Medical Rehabilitation, Obafemi Awolowo University, Ile-Ife, Nigeria; 3 Department of Nursing, University of Ibadan, Ibadan, Nigeria; 4 Department of Nursing Science, Obafemi Awolowo University, Ile-Ife, Nigeria; 5 Developmental Neurobiology and Forensic Anatomy Unit, Department of Anatomy, College of Medicine, University of Ibadan, Ibadan, Nigeria; 6 Department of Mental Health, Obafemi Awolowo University, Ile-Ife, Nigeria; 7 Department of Child Dental Health, Obafemi Awolowo University, Ile-Ife, Nigeria; 8 Oral Health Initiative, Nigerian Institute of Medical Research, Yaba, Lagos, Nigeria

**Keywords:** Sexual harassment, Victims, Survivors, Health facilities, Sexual abuse

## Abstract

**Background:**

Sexual assault occurring within healthcare settings represents a significant breach of public trust. This scoping review aimed to highlight the profile of people raped, those who committed the rape within the health facilities, and the legal actions taken to resolved cases.

**Methods:**

Media-reported data on incidents of rape in healthcare settings were collected. The search was conducted in May and June 2023, focusing on English-language publications with accessible full texts. Reports that lacked information on the survivors or incidents that occurred outside of healthcare settings were excluded. Descriptive statistics were used to summarize the categories of the collected publications, and graphical representations were employed for visualization purposes.

**Results:**

A total of 62 cases were retrieved, originating from Africa (*n* = 17; 27.4%), Europe (*n* = 14; 22.6%), Southeast Asia (*n* = 14; 22.6%), the Americas (*n* = 11; 17.7%), the Western Pacific Region (*n* = 5; 8.1%) and Eastern Mediterranean region (*n* = 1; 1.6%). In addition, 69 individuals were implicated in 59 cases. They were 31 doctors (44.9%), 17 (24.6%) nurses, four (5.8%) nurse/healthcare assistants, three (4.3%) cleaners/ward boy, two (2.9%) traditional medical doctors, and two (2.9%) security guards. Others included six (8.7%) staff members without designations and one (1.4%) ambulance driver. All perpetrators were male, ranging in age from 22 to 67 years. There were 66 victims identified in the 62 cases with age ranging from 2 to 92 years. Except for one case, all victims were female, and all but one case were patients. Most assaults occurred in consulting rooms/clinics (*n* = 21; 31.8%), 16 (24.2%) happened under sedation, and six (9.1%) were repeatedly raped, Survivors typically reported the cases the police (*n* = 12; 19.4%), family/friends (*n* = 11; 17.7%) or to hospital authorities (*n* = 10; 16.1%). Out of the 69 perpetrators, 19 (30.6%) were imprisoned with sentences ranging from 12 months to an indefinite period and one (1.6%) received a death sentence.

**Conclusion:**

The raping of patients by healthcare providers within healthcare settings calls for urgent and extensive measures. Stakeholders in healthcare management need to prioritize raising awareness about the problem, implement robust prevention and reporting strategies, and create healthcare environments that are safe, respectful, and supportive for all individuals seeking care.

**Supplementary Information:**

The online version contains supplementary material available at 10.1186/s40359-024-01721-w.

## Introduction

Rape is a grave violation of human rights, causing profound physical, psychological, and emotional harm to survivors [1–3]. While it is a widely recognized issue in society, there is little known about rape committed within healthcare settings. Healthcare settings, expected to provide care, support, and healing, can become unsafe for patients and staff due to the power dynamics and vulnerabilities inherent in the patient-provider relationship [4, 5]. This power dynamic can be exacerbated when patients are in vulnerable states, such as during examinations, procedures, or while under anaesthesia [6]. In addition, inadequate institutional policies and lack of comprehensive training on preventing and addressing sexual assault contribute to a culture of silence and impunity [6] within the healthcare setting. Furthermore, the absence of standardized reporting mechanisms and support systems for survivors also perpetuates a cycle of victimization and further emboldens perpetrators [6].

Sexual abuse committed by healthcare professionals has significant implications for victims and the healthcare system [7, 8]. It is an unwelcome traumatic experience that can leave long-term physical and psychological effects making affected individuals feel humiliated, intimidated, or uncomfortable [9], and may trigger or exacerbate serious mental health disorders [10]. It also undermines the integrity of the healthcare system, and tarnish the reputation of the medical profession, potentially discouraging individuals from seeking necessary medical attention [11]. This, in turn, can have a detrimental impact on public health outcomes, as delayed or avoided medical care can exacerbate existing conditions and lead to negative health consequences [12].

In addition, the perpetration of sexual abuse by healthcare professionals represents a profound breach of trust and an egregious violation of professional ethics. It undermines the foundational principles of medical ethics, including beneficence, non-maleficence, and respect for patient autonomy [13, 14]. Although comprehensive data on this specific form of sexual assault is limited, numerous studies and anecdotal evidence highlight its existence [15].

While sexual abuse in healthcare setting has been recognised as an occupational hazard for healthcare professionals perpetuated by patients, patients’ families, peers, and visitors [16, 17], there is little systematic documentation or comprehensive study about patients’ experience of sexual abuse within the healthcare setting. The extent of the problem of sexual abuse in healthcare institutions perpetuated by healthcare providers and allies working in the healthcare setting may be larger than recognised. This lack of data makes it difficult to fully understand the scale and scope of the problem; and how to systematically address the problem.

We, however, hypothesised using the feminist theory that rape within healthcare settings is not only a violation of individual rights but also a manifestation of broader power dynamics and inequalities entrenched within patriarchal society. Feminist theory emphasizes that power is unequally distributed along gender lines, contributing to the vulnerability of individuals, particularly women, within institutional settings. The patient-provider relationship, characterized by inherent power differentials, mirrors broader societal structures where women are often marginalized and disempowered. This power dynamic is exacerbated when patients, already in vulnerable states, are subjected to sexual violence by healthcare professionals who abuse their authority.

This aim of this scoping review, therefore, was to identify and synthesize the electronic media reports on rape of patients in healthcare institutions; and to analyse the cases for any observable pattern using relevant theoretical concepts. Specifically, the study highlighted the profile of people raped and those who committed the rape within the health facilities, who reported the case, pattern of how and where rape happened within the healthcare setting, and the legal actions taken to resolved cases.

## Methods

The proposed scoping review was guided by the Preferred Reporting Items for Systematic Reviews and Meta-Analyses Extension for Scoping Reviews (PRISMA-ScR) guidelines [18]. We developed our research questions, identified relevant studies using develop search strategies and following the inclusion and exclusion criteria, charted and collated the data, synthesised and developed the results.

### Research questions

This scoping review was guided by the questions (1) what is the profile of the survivors raped within healthcare facilities? (2) what is the profile of the perpetuators of rape within healthcare facilities? (3) who reported the cases of rape? and (4) what legal responses were provided?

### Search strategy

The search was conducted on the internet in between May and June 2023. Supplementary search was conducted in PubMed, Web of Science and Scopus. The search was conducted using the following key terms: “doctor” or “nurses” or “health attendants” or “health workers” and “rape” or “assault” and “patients” and “hospitals” or “clinic”. Search terms were updated according to the requirements of each electronic database where required. Details of search strings are accessible in Supplemental File 1.

### Eligibility criteria and selection

Literature obtained were exported into the reference management software Mendeley version 1.19.8. Duplicate articles were removed. Title and news content screening were performed independently by two researchers (AN and AI) using pre-defined inclusion and exclusion eligibility criteria. Uncertainty regarding whether publications met the inclusion criteria was resolved through consensus with one of the team members (MOF). No author or institution was contacted to verify the source of information.

### Inclusion criteria

Published reports written in English were identified through the searches. Only cases of rape that happened in healthcare settings were extracted. Rape was defined as “The penetration, no matter how slight, of the vagina or anus with any body part or object, or oral penetration by a sex organ of another person, without the consent of the victim.” [19, 20].

### Exclusion criteria

Cases were excluded if there were no profiling of the rape survivor, and if the reports of rape did not take place within a medical care facility.

### Data charting

The characteristics of studies including in the review are summarised in Table 1. The details on publishing media house, year of publication, location of the case, were extracted and reported. Other information summarised were the age, sex and status of the rape survivor, the age and sex and profession of the perpetuator, country where rape happened, who reported the case and the legal status of the case.

**Table 1:** Characteristics of the studies included in the scoping review (*n* = 62)

S/N	Author/ Publishing Media House (Year of publication) Source	Year rape happened	Country of reported case	Did the survivor report the case	Age/ Sex/ Profession of Perpetrator	Age/ Sex/ Status of Survivor	Where did rape happen?	Legal status of case
	African region							
1	Mathias Haufiku/ New Era (2013) https://allafrica.com/stories//201310041569.html	2013	Namibia	No information	25/M/Nurse	17/F/patient	No information	Arraigned in court
2	NA/PM news (2013) https://pmnewsnigeria.com/2013/10/24/nigerian-medical-doctor-arrested-for-raping-patient/?amp=1#aoh=16840681687321&referrer=https%3A%2F%2Fwww.google.com&amp_tf=From%20%251%24s&ampshare=https%3A%2F%2Fpmnewsnigeria.com%2F2013%2F10%2F24%2Fnigerian-medical-doctor-arrested-for-raping-patient%2F	2013	Nigeria	No information	NA/ NA/ Doctor	NA/F/patient	Ward	To be arraigned in court.
3	NA/South African Government (2013) https://www.gov.za/alleged-rape-patient-elizabeth-ross-hospital	2013	South Africa	Hospital authority	NA/M/Nurse	NA/F/patient	Ward	Information not available
4	Chioma Igbokwe/The Sun (2016) https://sunnewsonline.com/fertility-test-goes-messy-housewife-accuses-doctor-of-rape/	2016	Nigeria	Police	NA/M/Medico traditional doctor	NA/F/patient	Ultrasound room	Information not available
5	Danielle Ogbeche /Daily post (2016) https://dailypost.ng/2016/05/12/how-doctor-raped-impregnated-ulcer-patient-in-lagos/	2016	Nigeria	Police	NA/M/ Medico traditional doctor	26/ F/patient	Consulting room	Arrested
6	Christine Chihame /Zambia Daily Mail Limited (2017) http://www.daily-mail.co.zm/mansa-nurse-jailed-15-years-for-rape/	2016	Zambia	No information	35/M/Nurse	17/F/patient	Sedated and raped in emergency room	Imprisonment for 15 years with hard labour
7	Jenni Evans/ News 24 (2020) https://www.news24.com/news24/SouthAfrica/News/limpopo-doctor-jailed-for-8-years-for-raping-teenage-patient-on-examination-bed-20201103	2017	South Africa	To mother	55/M/Doctor	17/F/patient	Consulting room	Eight years imprisonment
8	Kenya Kambowe /Namibian Sun (2018) https://www.namibiansun.com/news/nurse-charged-with-hospital-rape2018-12-21	2018	Namibia	Hospital staff	NA/M/Nurse	56/ F/patient	No information	In police custody and denied bail (arrested)
9	NA /BBC News, Pidgin (2018) https://www.bbc.com/pidgin/tori-43701393	2018	Ghana	Family and the police	NA/M/Nurse	-	Ward	Got a reprimand from the court
10	Jaafar Jaafar /Daily Nigerian (2019) https://dailynigerian.com/exclusive-how-doctor-sedated-raped-married-patient-in-sokoto/	2019	Nigeria	Husband. Family lawyer involved the police.	NA/M/Doctor	14/ F/patient	Sedated and raped in consulting room	Information not available
11	NA /Sahara Reporters (2020) https://saharareporters.com/2020/11/12/police-arraign-medical-doctor-allegedly-raping-married-woman-adamawa	2020	Nigeria	Police	NA/M/Doctor	NA/F/patient	Consulting room	Arraigned in court
12	Nsikak Nseye /Dailypost.ng (2020) https://dailypost.ng/2020/07/08/two-year-old-raped-in-covid-19-hospital/	2020	South Africa	No information	Unknown	2/F/patient	COVID-19 isolation ward	Perpetrator not found
13	Lloyd M’bwana /The Marabi Post (2020) https://www.maravipost.com/shocking-south-africa-male-patient-raped-at-gauteng-hospital/	2020	South Africa	No information	Unknown	NA/M/patient	Psychiatry hospital	Ongoing investigation
14	NA/ Rédaction Africanews (2023) https://www.africanews.com/2023/04/05/guinea-doctors-convicted-for-rape-and-death-of-young-woman//	2021	Guinea	No information	Four Doctors	25/F/patient	Hospital. Battered to death	One doctor: 1 year imprisonment Two doctors: 15 years imprisonment One doctor: 20 years imprisonment
15	Malibongwe Dayimani/ News 24 (2022) https://www.news24.com/news24/southafrica/news/woman-allegedly-raped-by-medical-practitioner-at-eastern-cape-hospital-20221209	2022	South Africa	To relative who reported to police	39/M/Doctor	24/ F/patient	Consulting room	On the run
16	NA/Times Live (2022) https://www.timeslive.co.za/news/south-africa/2022-12-14-doctor-charged-with-rape-of-patient-at-hospital-consulting-rooms/	Undisclosed	South Africa	Nurse and friend	34/M/Doctor	34/F/patient	Consulting room	The doctor remained in custody until the court postponed the case to the following week for a formal bail application.
17	Tunde Oyekola /Punch Newspaper/(2023) https://punchng.com/doctor-rapes-nurse-in-kwara-suspect-remanded/	2023	Nigeria	Police	NA/M/Doctor	NA/F/patient	In theatre under anaesthesia	Remanded at a Federal correctional facility
	East Mediterranean region							
1	NA/Dunya News (2016) https://archive.pakistantoday.com.pk/2016/04/11/male-nurse-rapes-20-year-old-girl-patient-at-pimss-icu/	2016	Pakistan	Mother reported to hospital authority	NA/M/Nurse	20/F/patient with disability	Intensive Care Unit	Ongoing investigation and services of nurse terminated
	European region							
1	NA/ Deutsche Welle (2020) https://www.dw.com/en/french-doctor-on-trial-for-sexually-abusing-hundreds-of-children/a-52754167	1993	France	Child reported to parent in 2017	69/M/Doctor	4/F/patient	Hospital	Detained and arraigned in court
2	Lawrence Mass / Associated Press News (1999) https://apnews.com/article/8f9c74d9ce9bd122e40497ffec15e177	1998	UK	Nursing home authority	37/M/Nurse assistant	24/F/unconscious patient	Nursing home	Detained and arraigned in court
3	Sue Clough / The Telegraph (2000) https://www.telegraph.co.uk/news/uknews/1347355/Nurse-who-used-drug-to-rape-and-kill-gets-seven-life-sentences.html	1999	UK	Police	38/Nurse/M	33/F/patient	Accident and emergency.	Imprisonment of seven life terms.
4	NA/ BBC News (2010) https://www.bbc.com/news/uk-england-oxfordshire-11914580	2004	UK	Police	37/M/Nurse	17/F/patient	Side room of accident and emergency department	Imprisonment of 9 years
5	NA/ BBC News (2010) https://www.bbc.com/news/10419448	2008	UK	No information	59/M/Doctor	NA/F/patient with disability	Hospital	Jailed indefinitely
6	NA/ WSB-TV News (2010) https://www.wsbtv.com/news/athens-doctor-accused-of-drugging-raping-patient/241836107/	2009	Greece.	Police	52/M/Doctor	NA/F/patient	Sedated and raped in consulting room	Five years on probation and forbidden from treating female patients while on probation
7	Wesley Johnson/ The Telegraph (2013) https://www.telegraph.co.uk/news/uknews/crime/9799064/Healthcare-assistant-charged-over-rape-of-woman-in-hospital-bed.html	2013	UK	Hospital staff	29/ M/Healthcare assistant	NA/F/patient	Ward	Four count charge of rape and sexual assault
8	William Turvill/ Mail Online (2014) https://www.dailymail.co.uk/news/article-2538445/Mental-health-patient-tells-raped-60-times-care-worker-condemns-psychiatric-hospitals-playground-predators.html	Not reported	UK	No information	Senior staff member	NA/F/mental health patient	Repeated rape in ward	Imprisonment of 12-month, two-year suspension, paid £100,000 compensation in 2003 and 2004.
9	Eilish O’Regan / Irish Independent News (2016) https://www.independent.ie/irish-news/medic-accused-of-raping-patient-61-flees-country/35059432.html	2016	UK	Police	NA/M/Doctor	61/F/patient	Radiology changing room	Suspended by employer, resigned, and left the country (on the run)
10	Helen Lyons/ The Brussels Times (2021) https://www.brusselstimes.com/news/belgium-all-news/156899/brussels-gynecologist-who-raped-patient-gets-4-years-in-prison	2016	Belgium	Police	NA/M/Doctor	Early 20s/F/patient	Consulting room	4 years imprisonment
11	Ed Wight/ Mail online (2017) https://www.dailymail.co.uk/news/article-4898382/Boob-job-doctor-raped-patient-breast-surgery.html	2017	Samara, Russia	Local media	NA/M/Doctor	31/F/patient	Recovery room under anaesthetic sedation	Faces up to 6 years in jail
12	Darren Boyle/ Mail Online (2018) https://www.dailymail.co.uk/news/article-5922249/Mental-health-nurse-raped-patient-hospital-bed-jailed-15-years.html	2017	UK	Police	47/M/Nurse	NA/NA/patient	Sedated and raped in ward	Imprisonment of 15 years.
13	NA/BBC News (2020) https://www.bbc.com/news/world-europe-53597178	2020	UK	No information	52/ M/Healthcare assistant	73/F/client	Nursing home	11-year sentence
14	NA/The Guardian (2015) https://www.theguardian.com/uk-news/2015/apr/27/nurse-raped-unconscious-patients-hospital-jailed-18-years	Undisclosed	UK	Discovered video of incidences through a house search	29/M/Nurse	18/F/patient 35/F/patient Both were unconscious	Accident and emergency	Imprisonment of 18 years.
	The Americas regions							
1	NA/New York Times (1989) https://www.nytimes.com/1989/03/09/nyregion/hospital-aide-charged-in-rape-of-patient.html	1989	USA	No information	22/M/ Hospital cleaning aide 33/M/Security guard	38/F/patient	Psychiatry unit	Charged with third-degree rape and third-degree sexual assault. Charged with first-degree rape and third-degree sexual abuse.
2	NA/NYS AG’s Office (2000) https://ag.ny.gov/press-release/2000/oneida-nursing-home-aide-convicted-rape-92-year-old-patient	1997	USA	No information	NA/M/Nurse	92/F/client	Repeated rape in nursing home	Five counts of forcible rape in the first degree.
3	NA/The Washington Post (2013) https://www.washingtonpost.com/news/true-crime/wp/2018/08/19/a-jury-convicted-a-doctor-of-raping-a-patient-at-a-hospital-and-sentenced-him-to-probation/	2013	USA	Hospital authority	46/M/Doctor	NA/F/patient attached to ventilating machine	Ward	A decade of probation
4	NA/ABC News (2017) https://abc7ny.com/nurse-rape-alleged-raped-patient/1826944/	2017	USA	Female carer	27/M/Nurse	NA/F/Incapacitated patient	Ward	Charged with sexual assault.
5	NA/CNN (2017) https://edition.cnn.com/2017/08/20/health/nursing-home-aide-rape-trial-guilty/index.html#:~:text=Afteramp;20aamp;20weeklongamp;20trialamp;2amp;Camp;20Gomez,andamp;20isamp;20appealingamp;20theamp;20verdict	2017	USA	Hospital authority	56/M/Nurse Assistant	53/F/client 54/F/client	Nursing home	23 years imprisonment
6	NA/NA/AZ Central (2019) https://www.azcentral.com/story/news/local/phoenix/2019/01/23/hacienda-healthcare-sexual-assault-case-who-nathan-sutherland-nurse/2659072002/	2019	USA	Not applicable	36/ M/Nurse	29/ F/incapacitated client	Nursing home	10 years imprisonment
7	NA/CBC News (1999) https://www.cbc.ca/news/canada/sask-doctor-sentenced-for-rape-1.190253#:~:text=Aamp;20judgeamp;20inamp;20Saskatchewanamp;20has,aamp;20judgeamp;20inamp;20Reginaamp;20Thursday	1992	Canada	No information	NA/M/Doctor	23/F/patient	Sedated and raped in consulting room	Six years imprisonment
8	NA/Toronto star (2012) https://www.thestar.com/news/canada/2012/10/17/nursinghome_worker_jailed_12_months_for_sex_assault.html	2012	Canada	Not applicable	46/M/Nurse	71/F/client with severe dementia	Nursing home	12 months imprisonment
9	NA/Loop news (2022) https://cayman.loopnews.com/content/doctor-arrested-sexual-assault-pregnant-woman-during-c-section	2022	Brazil	Not applicable Caught on camera by suspecting colleagues	32/ M/Doctor	Undisclosed	In theatre under anaesthesia	Arrested and indicted for rape.
10	NA/Jamaica Star (2019) http://jamaica-star.com/article/news/20190906/15-y-o-patient-allegedly-raped-doctor	2019	Jamaica	Mother who reported to police	NA/M/Doctor	15/ F/patient	Consulting room	Under investigation and doctor on the run
11	NA/Radio Jamaica News (2023) http://radiojamaicanewsonline.com/local/autistic-teen-patient-allegedly-raped-at-kph	2023	Jamaica	Mother who reported to hospital authority	NA/M/Contract staff	17/F/Autistic patient	Hospital	Under investigation
	South-East Asia region							
1	NA/ BBC News (2015) https://www.bbc.com/news/world-asia-india-32793520 Aruna Shanbaug case - Wikipedia	1973	India	Not applicable	NA/M/Sweeper	25/F/Nurse	Raped and strangulated in clothe changing room	Seven years imprisonment
2	Lakmal Sooriyagoda / Daily Mirror (2022) https://www.dailymirror.lk/print/front_page/Negombo-Hospital-murder-case-CA-affirms-death-sentence-for-the-Doctor/238-239271	2007	Sri Lanka	Not applicable	NA/M/Doctor	23/F/patient	Raped, in consulting room, strangulated and let down the building while unconscious	Sentenced to death
3	Subhangi Kumari Singh/ Zee News (2019) https://zeenews.india.com/india/mumbai-doctor-rapes-blackmails-27-yr-old-circulates-objectionable-video-2240061.html	2015	India	Police	58/M/Doctor	27/F/patient	Sedated, repeated rape in consulting room and blackmailed.	Information not available
4	Rohit Bhan/ NDTV (2016 https://www.ndtv.com/india-news/gujarat-doctor-arrested-for-allegedly-raping-dengue-patient-1456501	2016	India	Uncle	28/M/Doctor and a ward boy	21/F/patient	Under sedation in the intensive care unit	Arrested but no further information available
5	Imran Fazal/ DNA (2016) https://www.dnaindia.com/mumbai/report-doctor-rapes-patient-inside-clinic-arrested-2236206	2016	India	Husband and the police	38/M/Doctor	29/F/patient	Consulting room	Arrested and booked but no further information available
6	Debashish Sarkar/ Hindu Times (2016) https://www.hindustantimes.com/india/jharkhand-hospital-head-sacked-after-guard-rapes-minor-rape-victim/story-5s4DOBy9s73eNGioFMLszH.html#:~:text=undergoingamp;20treatmentamp;20there.-,Theamp;20Jharkhandamp;20governmentamp;20onamp;20Tuesdayamp;20removedamp;20theamp;20superintendentamp;20ofamp;20Mahatma,treatmentamp;20thereamp;2Camp;20topamp;20officialsamp;20said	2016	India	No information	56/M/Security Guard	13/F/patient who is a rape survivor	Hospital	Ongoing interrogation
7	NA/PTI, The Indian Express (2018) https://indianexpress.com/article/india/muzaffarnagar-13-year-old-confined-raped-for-three-days-by-doctor-in-clinic-5147113/	2018	India	Father who reported to police	NA/ M/Doctor	13/F/patient	Sedated, confined for three days and raped in clinic	No information available
8	NA/New Age (2022) https://www.newagebd.net/article/183920/doctor-held-for-raping-patient-promising-marriage	2018	Bangladesh	No information	NA/M/Doctor	NA/F/ psychiatric patient	Repeated rape in clinic	Arrested and awaiting trial
9	NA/The times of India (2022) http://timesofindia.indiatimes.com/articleshow/92735627.cms?utm_source=contentofinterest&utm_medium=text&utm_campaign=cppst https://timesofindia.indiatimes.com/city/pune/pune-doctor-held-on-charge-of-raping-teen-patient/articleshow/92735627.cms?utm_source=contentofinterest&utm_medium=text&utm_campaign=cpps	2019	India	Police	44/M/Doctor	19/F/patient	Repeated rape in clinic and blackmail	No information
10	Rachael Bunyan / Mail Online (2020) https://www.dailymail.co.uk/news/article-8908857/Hospital-patient-20-gang-raped-doctor-medical-staff-murdered-India.html	2020	India	Not applicable	NA/M/Doctor and staff members	20/ F/patient	Gang raped in hospital and murdered.	Ongoing investigation Perpetuators on the run
11	Shaju Philip/ The Indian Express (2020) https://indianexpress.com/article/india/kerala/woman-who-tested-covid-positive-raped-by-ambulance-driver-on-way-to-hospital-in-kerala-6585068/	2020	India	No information	24/M/Ambulance driver	19/F/patient	In ambulance on way to COVID-19 isolation centre	Arrested. Dismissed from work.
12	NA /Antigua Newsroom (2021) https://antiguanewsroom.com/covid-patient-gang-raped-at-indian-hospital/	2021	India	Not applicable	Undisclosed three employees of the hospital	45/F/patient	Gang raped in COVID-19 isolation ward while on ventilator support before she died	Case being investigated
13	Anurag Dwary/ NDTV (2021) https://www.ndtv.com/india-news/covid-patient-raped-by-nurse-in-bhopal-hospital-died-in-24-hours-police-2441323	2021	India	A doctor	40/M/Nurse	43/ F/patient	COVID-19 isolation ward but died	Arrested and awaiting trial
14	NA/ Daily Mirror Online (2022) https://www.dailymirror.lk/breaking_news/Doctor-allegedly-sexually-abuses-patient-in-scan-room/108-244700	2022	Sri Lanka	Nurse	Undisclosed	15/F/patient	Raped in the scan room.	Medical officer assessed and ruled out rape. Investigation continuing.
	West Pacific region							
1	Tiffanie Turnbull/BBC (2022) Australiahttps://www.bbc.com/news/world-australia-62173774	2012	Australia	No information	58/M/Nurse	11/F/patient	Hospital	Accused committed suicide
2	Wan Ting Koh/Yahoo News (2018) https://sg.news.yahoo.com/general-practitioner-trial-alleged-rape-molest-23-year-old-student-050004600.html	2015	Singapore	Mother	67/M/Doctor	23/F/patient	Consulting room	On trial and faces up to 20 years’ jail and a fine
3	Kerry Kolasa-Sikiaridi/ Greek Reporter (2019) https://greekreporter.com/2019/01/08/former-gp-kyriakou-calls-rape-charges-stupid/	2016	Australia	Police	75/M/Doctor	61/F/patient 52/F/patient 49/F/patient	Clinic Clinic Clinic	Information not available
4	David Weber/ Australian Broadcasting Corporation (2019) https://www.abc.net.au/news/2019-06-21/doctor-sentenced-over-sex-assault-at-bunbury-regional-hospital/11232966	2017	Australia	No information	49/M/Doctor	45/F/patient	Consulting room	3 years imprisonment
5	Wan Ting Koh/Asia Times (2019) https://asiatimes.com/2019/02/gynecologist-charged-with-raping-sedated-indonesian-patient/	2018	Taiwan	No information	61/M/Doctor	30/F/pregnant patient	Theatre under general anaesthesia	No information

### Data analysis

Descriptive statistics, specifically frequencies and percentages, were used to present data. The countries where the cases were reported were classified by WHO region [21] into the Americas Regions (AMR); Eastern Mediterranean Region (EMR); African Region (AFR), European Region (EUR); Southeast Asian Region (SEAR) and the Western Pacific Region (WPR).

### Role of the funding source

The study was funded by out-of-pocket expenses. This had no role to play in the study design, data collection and analysis, decision to publish, or preparation of the manuscript.

## Results

**Fig. 1:**
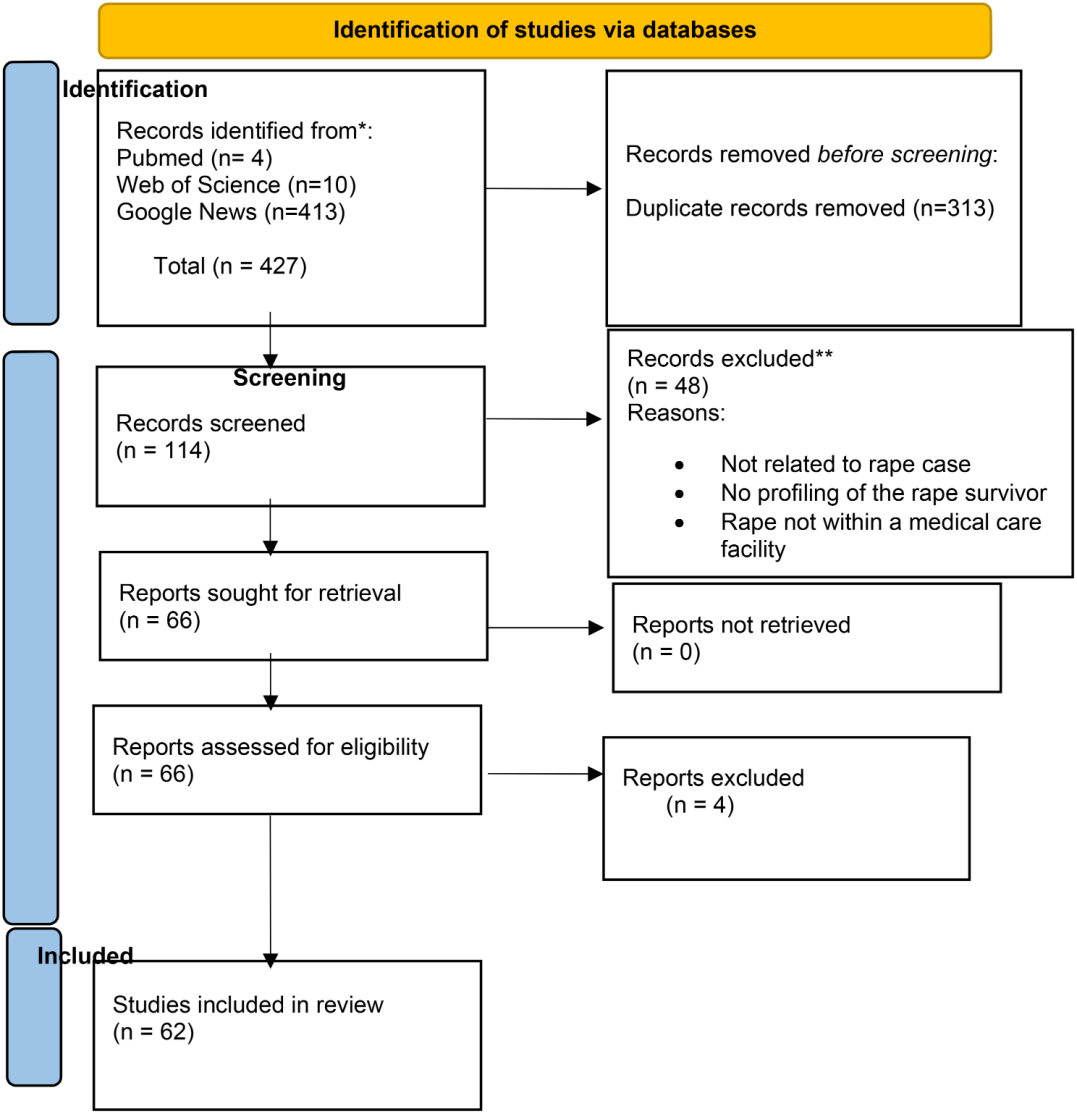
Flowchart of selection of media publications accessible on the internet, of cases of rape in healthcare facilities. *From* Page MJ, McKenzie JE, Bossuyt PM, Boutron I, Hoffmann TC, Mulrow CD, et al. The PRISMA 2020 statement: an updated guideline for reporting systematic reviews. BMJ 2021;372:n71. doi: 10.1136/bmj.n71

As Fig. 1 indicates, the initial search from 427 reports yielded 114 potentially relevant case reports. Overall, 52 publications were excluded based on the exclusion criteria leaving 62 reports for inclusion in the analysis.

### Distribution of cases

Figure 1 shows the distribution of the 62 reports by region while Table 1 shows the country where the incidence happened. The largest number of reports were from the AFR (27.4%)– six from Nigeria, six from South Africa, two from Namibia, and 1 from Ghana, Guinea, and Zambia respectively. This was followed by 14 (22.6%) reports from EUR– 10 from the United Kingdom and one case each from France, Greece, Belgium, and Russia; and 14 (22.6%) reports from the SEAR– 11 from India, two from Sri Lanka and one from Bangladesh. There were 11 (17.7%) reports from AMR– six from the United States of America, two from Canada and Jamaica respectively, and one from Brazil. There were five (8.1%) reports from WPR– three from Australia, one from Singapore and one from Taiwan. The least number of reports was from the EMR (1.6%)– the single report was from Pakistan.

### Temporal analysis

**Fig. 2:**
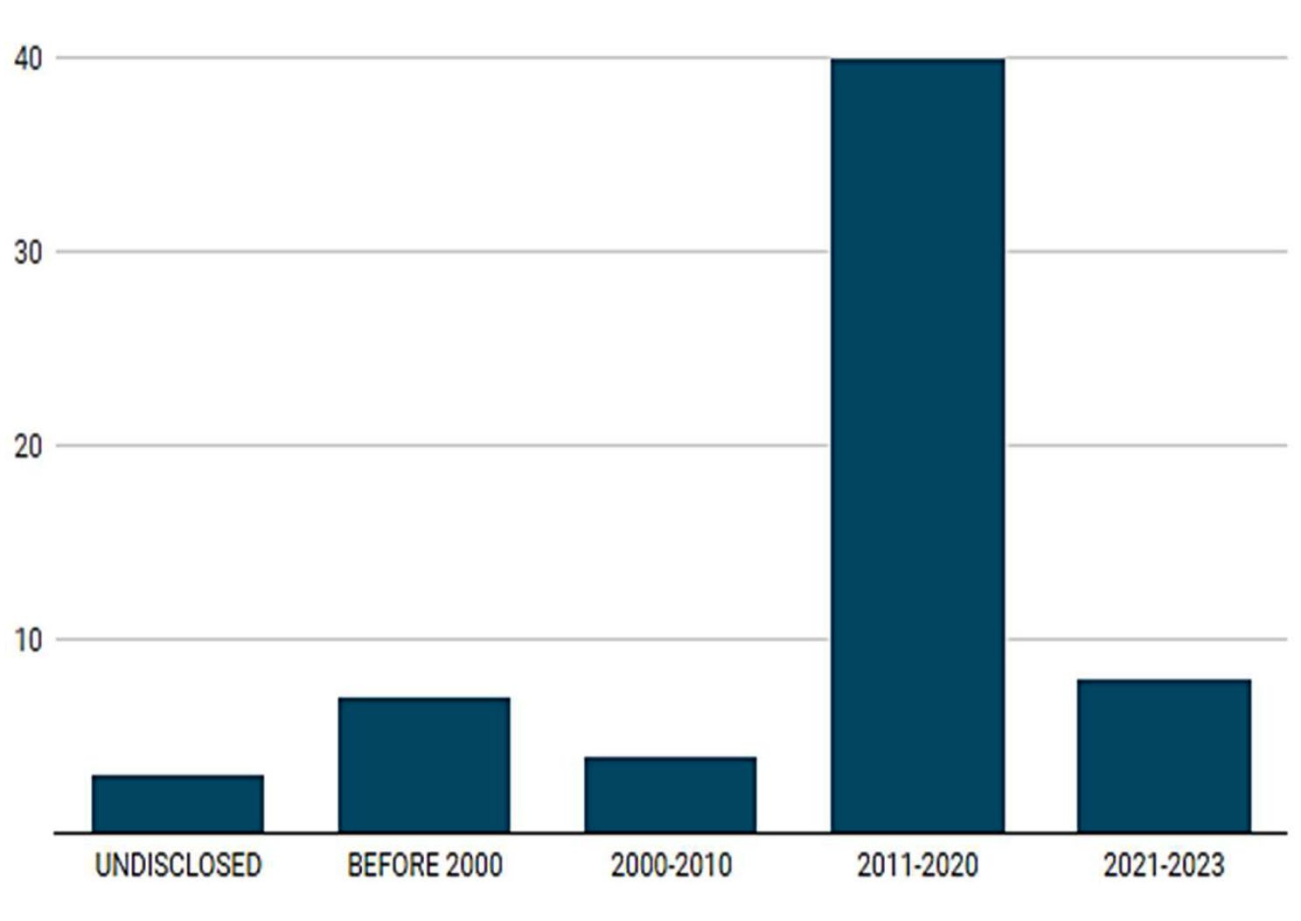
Year of reporting of the 62 incidences of rape in healthcare institutions

Table 1 shows that the first report dated back to 1973 in India. The most recent cases were reported in 2023– one in AFR (Nigeria) and AMR (Jamaica) respectively. Figure 2 shows that there were seven (11.3%) reports before 2000, four (6.5%) between 2000 and 2010, 40 (64.5%) between 2011 and 2020, and eight (12.9%) cases in the 3 years after 2020. Three (4.8%) of the 62 reports had undisclosed dates of incidence.

### Characteristics of perpetrators

Table 1 shows that different personnel in the healthcare institution committed the rapes. Of the 62 cases report, three (4.8%) of the cases had unknown perpetuators, while the remaining 59 cases had 69 perpetuators. These were 31 doctors (44.9%), 17 (24.6%) nurses, four (5.8%) nurse/healthcare assistants, three (4.3%) cleaners/ward boy, two (2.9%) traditional medical doctors, and two (2.9%) security guards. Others included six (8.7%) staff members without designations and one (1.4%) ambulance driver. Four of the cases were gang rapes within the health institutions– one in Guinea and three in India.

Table 1 shows that all identified perpetuators were males. The ages of 36 of the 59 perpetuators identified ranged from 22 years to 67 years with a mean (standard deviation) age of 45.44 (13.97) years.

### Characteristics of the victims

As shown in Table 1, there were 66 persons involved in the 62 reported cases. Of these 21 (31.8%) reported rape occurred in the consulting room/clinics, eight (12.1%) occurred in the wards, six (9.1%) in nursing homes, four (6.1%) in the accident and emergency, four (6.1%) in theatre/recovery room, four (6.1%) in radiology/scan rooms, two (3.0%) in isolation wards, two (3.0%) in psychiatric hospital/homes, two (3.0%) in intensive care units, one (1.5%) in the ambulance (*n* = 1) and one (1.5%) in the nursing changing room. However, there were 11 cases where information about the location of the assault within the hospital could not be obtained.

In addition, 16 (24.2%) cases of rape happened under sedation– purposefully sedated by perpetuator, or while under general anesthesia. six (9.1%) were repeatedly raped, four (6.1%) died after been raped, two (3.0%) of the victims were blackmailed, and two (3.0%) were strangulated (one lived as a vegetable) after the rape.

The media report disclosed the ages of 46 victims, ranging from 2 years to 92 years, with a mean (standard deviation) age of 33.44 (23.39) years. Among the survivors, 15 were children and adolescents and five were elderlies above 60 years of age. All the victims were females, except for one male victim who experienced rape in a psychiatry hospital in South Africa. All the victims were also patients except for one female nurse who was raped in the nurses changing room in India. Patients molested include those with disability (*n* = 4), those with mental health challenges (*n* = 3), those unconscious (*n* = 3), those with communicable disease (*n* = 3), on ventilator (*n* = 1) and autistic (*n* = 1).

### Disclosure and Legal outcomes

Of the 62 cases, 18 (29.0%) reported the incidents of which 12 (19.4%) were reported to the police, 11 (17.7%) to family or friends, 10 (16.1%) to hospital authorities, and two (3.2%) to both the police and family. Seven (11.3%) cases could not have made reports because they were either killed, unconscious or had severe disabilities. One reported to the media (1.6%) and one (1.6%) was discovered through other investigations.

The legal outcomes of the 62 cases were as follows: one (1.6%) received a death sentence, 18 (29.0%) led to imprisonment with sentences ranging from 12 months to an indefinite period, two (3.2%) were placed on probation, one (1.6%) received a reprimand from the court, and three (4.8%) was convicted but no details about the length of the jail term were provided. Additionally, one (1.6%) case was awarded a compensation of £100,000 per year for 2 years. Regarding the status of the other perpetrators at the time of the media reports, eight (12.9%) had been arrested, seven (11.2%) had been charged and were awaiting trial, six (9.7%) were under ongoing investigations, three (4.8%) were fugitives, two (3.2%) was scheduled to be charged, one (1.6%) could not be located, and one (1.6%) had a preliminary determination of ‘no rape’ by the investigating medical officer. One perpetrator (1.6%) committed suicide a week after the allegation. Information on actions being taken against seven perpetrators (11.3%) was unavailable.

## Discussion

Our review of the scope of rape cases in healthcare facilities suggests that it is a global problem, perpetuated by health care workers and non-healthcare workers in healthcare facilities and nursing homes. Most of the perpetrators were male and middle-aged, utilizing their positions of authority as healthcare providers to gain access to the victims. Females are worse affected, and when reported, receive poor legal attention. Furthermore, the study revealed that only one out of four cases reported by the media resulted in a conclusive legal determination. This suggests that a significant number of cases did not reach a resolution at the time of media reporting.

This is the first study that undertook a review of cases of rape perpetuated in health facilities. The review, however, has a few limitations. First, the scope was limited to case reports written in English and those reports accessible on the internet. These criteria lead to the exclusion of some reports, including those reported to legal authorities but not captured by the media, and those reported in media outlets not accessible on the internet. In addition, the use of the search term ‘health workers’ may not have captured the diverse range of healthcare workers in the health sector. these gaps could have led to the under-reporting of the cases. Despite this risk of under-reporting, the review highlights a few critical findings.

First, health care settings are critical site for connecting survivors to care [22]. That rape happens within healthcare settings, that patients who need care for rape are raped within healthcare settings [23], and that healthcare providers are the major perpetuators is a major concern. The breach of trust by a trusted healthcare professional can lead to profound trauma, eroding their sense of safety and security. Survivors may experience mental health disorders such as post-traumatic stress disorder, anxiety, depression, and may also have difficulty seeking future medical care, often leading to delayed or neglected treatment [2, 7]. This finding highlights the need to identify systems related factors that enable such abuse; measures that can be instituted to prevent and break the culture of silence on rape of patients by healthcare workers and allies in health facilities; and systems that need to be put in place to reduce the risk of patients being exploited by those who work in the health care institutions.

The study findings indicates that abuse happens most frequently in the consulting room within healthcare settings. The consulting room is intended to be a safe and professional environment where patients seek medical care and treatment. The practice of doctors having chaperones when seeing patients should reduce this risk of rape [24, 25] though there were cases where the rape happened immediately after the chaperone left the office for official duties [26, 27]. The possibility for rapes to happen even when chaperones are designated to clinics, suggests the need for institution of strict practice protocols, enhancing staff training on ethical conduct and professional boundaries, promoting a culture of zero tolerance for abuse, and establishing effective mechanisms for identifying. reporting and addressing incidents and suspected cases of abuse by patients and staff.

One system for prompt identifying of cases is for hospitals to invest in the use of technologies that enables the non-intrusive monitoring of what goes on in the consulting rooms, wards, and theatres as an additional safeguard measure for patients. This does not preclude instituting systems and processes that promotes the culture of accountability among the staff in the hospital; the strengthening of policies and procedures that prevents sexual harassment of patients and the prompt reporting of cases to appropriate authorities; providing regular education and training to healthcare professionals; and supporting survivors with appropriate resources and services.

Second, we observe that victims of rape may turn to the family/friends, hospital authorities or the police as the person first notified. It is therefore important that the competencies of those often notified should be built to properly address cases, especially in ways which effectively support the healing of the survivor. Although perpetuators may want to settle cases out of court, they should face the civil or criminal justice systems in pursuit of legal justice on behalf of the victims, and as a deterrent for a repeat. Survivors also need informal support groups post-assault, to navigate the civil and criminal justice systems, cope with the traumatic experience they have endured, and in interpreting these experiences [28]. Furthermore, the legal systems need to be strengthened to ensure that survivors of sexual violence have access to justice and support services.

Public discussions and awareness of the possibility of sexual assaults and harassment in the confines of hospital, can also help to improve the judiciary system for handling these cases. Ensuring that perpetrators are held accountable for their actions through legal and disciplinary measures is also essential to prevent further instances of abuse and to restore trust in the healthcare system. It is also important to foster a culture that supports and believes survivors, while challenging societal attitudes that perpetuate victim-blaming and stigma [29].

Sexual abuse by health care professionals is very likely to be underreported because of factors such as fear, shame, power imbalances, and concerns about retaliation or not being believed [30, 31]. Often, the stigma of sexual abuse is on the survivor who having survived the abuse, is often unable to survive the social repercussions associated with reporting. Shame often leads to foregoing medical and legal help to avoid further humiliation [9]. In addition, survivors may also not realize that what was happening was wrong, illegal, or a form of abuse [32]. Furthermore, it is a lot more difficult to report sexual abuse perpetuated by persons in the position of power and trust like a healthcare provider. Healthcare workers are granted access to their patients’ bodies and should only use that access in a manner appropriate for medical care. Often, the onus then lies with the survivor to prove that the health professional’s access to the patient’s body was in a manner inappropriate for medical care. This abuse of power underscores the urgency of addressing the issue.

Third, we noticed a tremendous increase in the number of reported cases between 2011 and 2020 when compared to the previous decade, and a high rising number in the years after 2020. We feel this increase in the number of reports may be due to several factors, including heightened awareness and advocacy efforts, improved reporting mechanisms, changes in societal attitudes towards reporting, and possibly an actual increase in the incidence of the observed phenomena. Further studies are needed to understand this observation as the findings may help to strengthen public response to preventing and addressing the rape of patients in health institutions. In addition, future studies should also investigate the observed syndemic nature of rape within healthcare institutions. This encompasses not only instances of sexual assault against females but also the rape of females who are vulnerable such as those with disabilities, the elderly and sedated patients as highlighted in this study.

This study highlights that within the healthcare setting, there exist diverse populations of rapists who may exhibit distinct motivations and psychological characteristics [33]. We postulate that rapists in the healthcare sector represent a population with psychological traits that have received limited research attention. These individuals may demonstrate low self-control, impulsivity, a penchant for adventure, self-centeredness, and a tendency to engage in immediate gratification through criminal acts, as proposed by the self-control theory [34]. The use of sedatives by some healthcare rapists or the act of raping victims under general anaesthesia can be likened to using force to obtain sexual satisfaction, as described by the Narcissistic Reactance Theory [35]. However, it is important to note that the context in which rape occurs in the healthcare setting does not align with the Narcissistic Reactance Theory’s premise that forceful sex is a reaction to a refusal of sexual advances by the victim. Similarly, the actions of healthcare providers who commit rape do not align with the Feminist Theory, which posits that rape is a conscious process of intimidation aimed at keeping women in a state of fear [36]. In addition, the dynamics of rape within the healthcare setting do not completely support the idea of perpetuators wanting to instil fear in victims though the cases that used blackmail to keep their hold over the survivors to enable them to continue to rape, may suggest otherwise. Our findings suggest that healthcare workers who commit rape view their victims as easy targets for satisfying their sexual urges, with little regard for the health and well-being of the victims.

However, it is crucial to acknowledge the limitations of applying theoretical frameworks to the unique context of healthcare-related sexual abuse. While elements of self-control theory may offer insights into the motivations of perpetrators, the dynamics of power and vulnerability within healthcare settings complicate traditional understandings of rape as solely an expression of individual pathology or deviance. Moreover, the intersectionality of factors such as race, gender, age, and disability further shape the experiences of survivors and the responses of healthcare institutions. The events reported in this scoping review, however, suggests the lack of comprehensive institutional policies and training to address sexual assault. This reflects a systemic failure to prioritize the safety and well-being of survivors, further perpetuating a culture of silence and impunity. While the healthcare setting may appear safe in public, they pose a risk to vulnerable populations, as evidenced by the profile of the rape victims in this study.

Moving forward, it is imperative to address the systemic failures and institutional barriers that enable sexual abuse to occur within healthcare settings. Comprehensive policies and repeated trainings on ethical conduct and professional boundaries, need to be implemented to prevent and respond to instances of sexual violence, and prioritize survivor-centered approaches that promote safety, dignity, and justice. The policies also need to promote a culture of zero tolerance for abuse, and establish effective mechanisms for identifying, reporting, and addressing incidents of abuse by both patients and staff. Additionally, efforts to challenge societal attitudes and beliefs that perpetuate victim-blaming and stigma are essential to creating a culture of accountability and support for survivors to promptly report cases of abuse. Further research is needed to explore the intersection of gender, power, and healthcare-related sexual abuse, with a focus on validating and expanding upon the hypotheses generated by this study.

In conclusion, the incidence of rape of patients by healthcare providers within healthcare settings is a distressing occurrence that demands immediate attention [37, 38]. It is crucial that stakeholders involved with healthcare management and patients’ care acknowledge the existence of this issue, raise awareness, and implement comprehensive measures to prevent and address cases of sexual assault. By prioritizing survivor-centered approaches, robust institutional policies, and transformative cultural change, it is possible to create healthcare environments that are truly safe, respectful, and supportive for all individuals seeking care.

### Electronic supplementary material

Below is the link to the electronic supplementary material.

Supplementary Material 1

## Data Availability

The datasets used and/or analyzed during the current study are available from the corresponding author on reasonable request.

## Abbreviations

AFR: African Region
AMR: The Americas Regions
EMR: Eastern Mediterranean Region
EUR: European Region
SEAR: Southeast Asian Region
UK: United Kingdom
USA: United States of America
WPR: Western Pacific Region
