# Supplementary material for: Healers that hurt: a scoping review of media reports of cases of rape in healthcare settings
Source: BMC Psychol. 2024 Apr 16;12:210. doi: 10.1186/s40359-024-01721-w (PMC11020642; doi:10.1186/s40359-024-01721-w)
Supplement: Supplementary file 1 — Supplementary Material 1 [file 40359_2024_1721_MOESM1_ESM.docx]

Supplemental 1: Search strings and findings from databases

**Pubmed search strategies**

| # | Query | Results |
| --- | --- | --- |
| 16 | **(((((#1) AND (#2)) AND (#3)) AND (#4))) AND (#13)** | **0** |
| 15 | **(((((#1) AND (#2)) AND (#3)) AND (#4))) AND (#12)** | **0** |
| 14 | **(((((#1) AND (#2)) AND (#3)) AND (#4))) AND (#11)** | **1** |
| 13 | ("western pacific"[Title/Abstract]) | 2,689 |
| 12 | ("eastern mediterranean"[Title/Abstract]) | 3,193 |
| 11 | ("America"[Title/Abstract]) | 109,954 |
| **10** | **((((#1) AND (#2)) AND (#3)) AND (#4)) AND (#9)** | **0** |
| 9 | ("southeast asia"[Title/Abstract]) | 12,484 |
| **8** | #1 AND #2 AND #3 AND #4 AND #7 | **1** |
| 7 | ("Africa"[Title/Abstract]) | 143,033 |
| **6** | #1 AND #2 AND #3 AND #4 AND #5 | **2** |
| 5 | ("Europe"[Title/Abstract]) | 135,684 |
| 4 | ("hospitals"[Title/Abstract] OR "clinic"[Title/Abstract]) | 537,491 |
| 3 | ("patients"[Title/Abstract]) | 6,666,036 |
| 2 | ("rape"[Title/Abstract] OR "assault"[Title/Abstract]) | 19,784 |
| 1 | ("doctor"[Title/Abstract] OR "nurses"[Title/Abstract] OR "health attendants"[Title/Abstract] OR "health workers"[Title/Abstract]) AND (fha[Filter]) | 252,797 |

**Web of Science Search Strategies**

| 16 | #1 AND #2 AND #3 AND #4 AND #10 | 0 |
| --- | --- | --- |
| 15 | #1 AND #2 AND #3 AND #4 AND #9 | 0 |
| 14 | #1 AND #2 AND #3 AND #4 AND #8 | 0 |
| 13 | #1 AND #2 AND #3 AND #4 AND #7 | 1 |
| 12 | #1 AND #2 AND #3 AND #4 AND #6 | 8 |
| 11 | #1 AND #2 AND #3 AND #4 AND #5 | 1 |
| 10 | AB= (western pacific) | 25,870 |
| 9 | AB= (eastern mediterranean) | 15,337 |
| 8 | AB=(America) | 341,186 |
| 7 | **AB= (southeast asia)** | [22,567](https://0-www.webofscience.com.wam.seals.ac.za/wos/woscc/summary/16166fbd-14c5-47da-96f4-d74313b7bfe1-9a491973/relevance/1) |
| 6 | AB=(Africa) | 220,539 |
| 5 | **AB=(Europe)** | [245,869](https://0-www.webofscience.com.wam.seals.ac.za/wos/woscc/summary/d7c52ade-a20e-4753-92a0-f1a3a8391ad7-9a48da98/relevance/1) |
| 4 | (AB=(hospital)) OR AB=(clinic) | 1,223,216 |
| 3 | AB=(patient) | 5,764,307 |
| 2 | (AB=(rape)) OR AB=(assault) | 32,101 |
| 1 | (((AB=(doctor)) OR AB=(nurses)) OR AB=(health attendants)) OR AB=(health workers) | 400,851 |

**Google news search terms:** Doctor or nurses OR health attendants OR health worker AND rape or sexual assault AND Patient AND hospital or clinic

| **Regions** | **Results** |
| --- | --- |
| Europe | 98 |
| Africa | 100 |
| America | 92 |
| Southeast Asia | 34 |
| Eastern Mediterranean | 34 |
| Western pacific | 55 |
